# Characterization and functional analysis of zinc trafficking in the human fungal pathogen *Candida parapsilosis*

**DOI:** 10.1098/rsob.220077

**Published:** 2022-07-13

**Authors:** Tamás Takács, Mihály Tibor Németh, Flóra Bohner, Csaba Vágvölgyi, Ferenc Jankovics, Duncan Wilson, Attila Gácser

**Affiliations:** ^1^ HCEMM-USZ Fungal Pathogens Research Group, Department of Microbiology, Faculty of Science and Informatics, University of Szeged, Szeged, Hungary; ^2^ Department of Microbiology, Faculty of Science and Informatics, University of Szeged, Szeged, Hungary; ^3^ Department of Medical Biology, Szent-Györgyi Albert Medical School, University of Szeged, Szeged, Hungary; ^4^ MTA-SZTE ‘Lendület’ Mycobiome Research Group, University of Szeged, Szeged, Hungary; ^5^ Institute of Genetics, Biological Research Centre, Szeged, Hungary; ^6^ Medical Research Council Centre for Medical Mycology at the University of Exeter, Exeter, UK

**Keywords:** zinc, pathogen, *Candida*, macrophage

## Abstract

The zinc restriction and zinc toxicity are part of host defence, called nutritional immunity. The crucial role of zinc homeostasis in microbial survival within a host is established, but little is known about these processes in the opportunistic human fungal pathogen *Candida parapsilosis.* Our *in silico* predictions suggested the presence of at least six potential zinc transporters (ZnTs) in *C. parapsilosis*—orthologues of *ZRC1*, *ZRT3* and *ZRT101*—but an orthologue of *PRA1* zincophore was not found. In addition, we detected a species-specific gene expansion of the novel ZnT *ZRT2,* as we identified three orthologue genes in the genome of *C. parapsilosis*. Based on predictions, we created homozygous mutant strains of the potential ZnTs and characterized them. Despite the apparent gene expansion of *ZRT2* in *C. parapsilosis*, only *CpZRT21* was essential for growth in a zinc-depleted acidic environment, in addition we found that CpZrc1 is essential for zinc detoxification and also protects the fungi against the elimination of murine macrophages. Significantly, we demonstrated that *C. parapsilosis* forms zincosomes in a Zrc1-independent manner and zinc detoxification is mediated by the vacuolar importer CpZrc1. Our study defines the functions of *C. parapsilosis* ZnTs, including a species-specific survival and zinc detoxification system.

## Graphical abstract

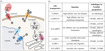

## 1. Introduction

Among trace elements, metal ions such as iron and zinc are vital for living organisms. These micronutrients are crucial for maintaining cellular homeostasis and approximately one-third of all proteins require metal cofactors to properly function. Metal elements are also essential for several metabolic processes such as DNA polymerase activity and transcription modifier mechanisms [1,2]. Zinc is the second most abundant trace metal after iron in humans. Zinc plays an important role in the function of both the innate and adaptive immune system, and it is required for the function of a wide variety of enzymes and other proteins including superoxide dismutases, alcohol dehydrogenases and transcription factors [3,4]. To survive within a mammalian host, human fungal pathogens use iron and zinc from the host's environment. This has been previously demonstrated by functional studies, where the absence of iron or zinc-acquisition-related factors (e.g. transporters and binding proteins) resulted in decreased virulence and growth defects of pathogenic fungi [5]. Limiting the accessibility of certain microelements is also an effective solution to control the growth of pathogens. For instance, the host is able to keep trace elements in a bound form using secreted metal-binding proteins, such as calprotectin, thereby limiting their uptake by pathogens, and thus inhibiting the extent of an infection [5]. On the other hand, zinc can also be toxic to pathogens. For example, murine macrophages are able to raise zinc concentration to a toxic level in the phagolysosome and effectively eliminate *Mycobacterium tuberculosis* in this compartment [6]. Furthermore, in the case of murine BMDMs, an IFN-I-dependent regulatory pathway can result in zinc toxification in the phagolysosome following *Candida glabrata* infection [7]. Taken together, pathogens face a ‘double-barrelled shotgun’ when encountering a potential host, as they need to overcome not only the limited accessibility of essential nutrients but, upon an effective host cellular response, potentially toxic levels of metal ions. These protective responses by the host are known as nutritional immunity [5].

In order to survive in the host, fungal pathogens require transport systems to access zinc ions from their environment as well as zinc detoxification machinery to protect against a deadly amount of zinc within phagolysosomes. Eukaryotic organisms encode multiple zinc transporters (ZnTs) to maintain cellular zinc homeostasis. Zrt-, Irt-like proteins (Zip) transporters transport zinc ions from the extracellular space to the cytoplasm [8], whereas ZnTs are responsible for the transport of zinc ions from the cytoplasm into intracellular organelles in the presence of high zinc. To date, the majority of information about fungal zinc homeostasis comes from Saccharomyces *cerevisiae*. In *S. cerevisiae*, several ZnT proteins (ScZrt1, ScZrt2, ScZrt3, ScZrc1 and ScFet4) have been described that, along with certain stress proteins, are regulated by the transcription factor Zap1 [9]. The *ScZRT1*, *ScZRT2* and *ScFET4* genes encode plasma membrane-localized transporters that are responsible for the transport of zinc ions from the environment to the cytoplasm. ScZrt1 acts as a high-affinity ZnT, while ScZrt2 and ScFet4 act as low-affinity transporters [9]. To prevent cellular toxicity, *S. cerevisiae* intracellular ZnTs actively transport accumulated zinc into a vacuole via ScZrc1 and ScCot1 [10]. Additionally, under zinc depletion, the vacuolar ScZrt3 transports zinc from the vacuole stores to the fungal cytoplasm [11].

Zinc uptake systems have also been studied in human fungal pathogens including *Aspergillus fumigatus*, *Blastomyces dermatitidis*, *Histoplasma capsulatum*, *Candida dubliniensis* and *C. albicans.* In *C. albicans*, the primary mode of uptake that has been described in this species takes place through the zincophore system, where the limited amount of zinc ions are bound by a zinc-binding protein or ‘zincophore’ [12]. The process is mediated by the secreted Pra1 (pH-regulated antigen 1) protein, which is able to bind zinc ions with high affinity in the host's environment and transport them to the fungal Zrt101 plasma membrane transporter. Citiulo *et al*. demonstrated that *C. albicans* can use zinc from the cytoplasm of endothelial cells when it is in the penetrating hyphae form [12]. In addition to the Pra1-Zrt101 synergic zinc uptake system, which functions at neutral–alkaline pH, *C. albicans* uses Zrt2 plasma membrane-located ZnTs to obtain zinc from acidic environments [13]. Crawford *et al*. showed that *CaZRT1* expression is pH-dependent and supports the growth of *C. albicans* cells in the absence of CaZrt2 at neutral–alkaline pH. The deletion of *CaZRT2* reduces fungal colonization of kidneys in mice [13]. *C. albicans* is able to store the accumulated zinc ions through a rapid compartmentalizing process into zincosomes, which is mediated by CaZrc1.

Over the past two decades, non-*albicans Candida* species have gained significant importance in medicine due to their increasing prevalence in life-threatening and frequently lethal infections. Of the non-*albicans Candida*, *C. parapsilosis* is often identified as the second or third most common cause of candidemia (5%–46%) and its prevalence is dependent on geographical regions and the examined patient groups [14]. This species particularly threatens low birth weight infants, the immunosuppressed and patients receiving long-term antibiotic treatment or chemotherapy [14]. While the main transmission route of *C. albicans* is thought to be vertical, with humans colonized at an early age (first months of life), the most common source of *C. parapsilosis* transmission is through the hands of healthcare workers or non-sterile clinical devices [15]. Several studies have reported that *C. parapsilosis* is less susceptible to echinocandins and resistance to azoles has also been reported [16,17]. *C. parapsilosis* also effectively adheres to and forms biofilms on catheters and other medical equipment [18]. Besides secreted hydrolytic enzyme and prostaglandin production, morphological switching with biofilm formation, metal ion acquisition has been suggested as an important prerequisite of invasion by this species [19–22].

Notably, perturbed iron homeostasis affects the virulence of *C. parapsilosis* [23,24]. Furthermore, the multi-copper oxidase encoded by *FET3* also influences the species' morphology, biofilm formation and prostaglandin production, all of which are confirmed *C. parapsilosis* virulence factors. Similar to other pathogens, other metals and their transporters may also aid the virulence of *C. parapsilosis*. Here, we examine the ZnTs of *C. parapsilosis in silico* and have generated homozygous knock-out mutants of each. We further aimed to expose the generated mutant strains to various types of stressors and zinc-limiting conditions and to define their function in zinc transport of *C. parapsilosis*. In this study, we also aimed to uncover their role in host–pathogen interactions using *in vitro* macrophage and *in vivo* infection models.

## 2. Material and methods

### 2.1. Strain maintenance and cultivation

All of the strains used in this study were stored frozen at −80°C. Homozygous mutants were maintained on selective drop out (0.19% YNB-2% D-glucose) agar media supplemented with 100 unit ml^−1^ penicillin-streptomycin. Heterozygous mutant strains were maintained similarly, with L-Leucine (2.5 mg ml^−1^) added to the media as a supplement. The *C. parapsilosis* CLIB 214 wild-type strain was maintained on YPD (1% D-glucose, 1% peptone, 0.5% yeast extract) media containing 100 unit ml^−1^ penicillin-streptomycin. The gene reintegrated and green fluorescence protein (GFP)-labelled strains were maintained on YPD supplemented with 200 µg ml^−1^ nourseothricin.

Prior to the experiments, strains were cultured in YPD, then 100 µl of the cultivation was re-inoculated in 2 ml of YPD liquid medium containing 100 unit ml^−1^ penicillin-streptomycin at 30°C with overnight shaking (200 r.p.m.). Cells were then washed with 1 × PBS three times before adjusting to the working concentrations.

### 2.2. Generation of *Candida parapsilosis* mutant strains

All strains used for this study are summarized in the electronic supplementary material, table S1. Primers used in this study are summarized in the electronic supplementary material, table S2, plasmids in the electronic supplementary material, table S3. All mutant strains were verified with colony PCR and Southern hybridization.

Hetero- and homozygous mutants were generated according to the protocol described by Holland *et al*., using the *C. parapsilosis* CLIB 214 histidine and leucine auxotrophic strain, with modifications in the transformation method [25]. Two individual transformants were selected for each gene.

For reintegration, *CpZRT21* and *CpZRC1* were introduced into the *CpNEUT5 L* neutral locus using the Gateway cloning method as adapted to *C. parapsilosis* by Németh *et al.* [26]. For functional restoration, *CpZRC1* with a *CaTDH3* promoter was introduced into the mutant strain Δ/Δ *CpZRC1* by transformation as described by Németh *et al.* [27].

For phagocytosis experiments, GFP-labelled CLIB 214 RI, Δ/Δ *CpZRT21* and Δ/Δ *CpZRC1* cells were used, where the *CaTDH3*-GFP fusion construction was integrated into the *CpNEUT5 L* neutral locus as described by Németh *et al.* [27].

To determine the intracellular localization of CpZrc1, we introduced Gly5-GFP on the C-terminus of the target gene according to the CRISPR-Cas9-based gene modification and transformation method described by Lombardi *et al.* [28].

### 2.3. Phenotypic characterization

Viability of each homozygous mutant was tested under the 30 different stress conditions listed in electronic supplementary material, table S3. Strains were prepared and washed as mentioned above. Cell concentration was adjusted to 10^4^/10³/10²/10 cells/ in 5 µl. The growth of each strain was examined after 3 days of incubation at 30 C. 4 × 10^5^ cells were plated into 96-well plates and growth kinetics was determined by spectrophotometry. Optical density was measured at 600 nm.

### 2.4. Low zinc medium

To characterize ZnT mutant strains growth properties and tolerance to zinc, we used low zinc medium (LZM) supplemented with different concentrations (0.05, 1, 10, 15, 17.5, 20, 22.5, 25, 50 and 100 mM) of zinc from 0.1 or 2 M ZnSO_4_ stock solutions. To prepare the base LZM, we created the medium by adding all essential amino acids, nutrients and ions except zinc. We followed a modified protocol provided by Crawford *et al.* [13]. The unbuffered pH of our LZM was 7.5. To adjust to acidic/alkalic pH, citric acid (1 M) or sodium phosphate (0.2 M) was used, respectively.

### 2.5. Gene expression analysis

**Figure 2.**
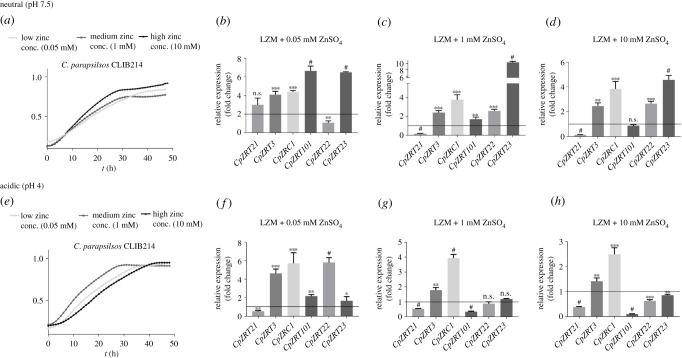
Gene expression changes in the expression of ZnTs under neutral (pH 7.5) and acidic (pH 4) conditions. *C. parapsilosis* CLIB 214 cells were grown in YPD, and then inoculated into liquid LZM with neutral or acidic pH supplemented with low (0.05 mM), medium (1 mM) and high (10 mM) concentrations of ZnSO^4^. (*a*,*d*) Growth kinetics of *C. parapsilosis* CLIB 214 cells in different zinc-supplemented LZM media. (*b*–*d*, *e–g*) Gene expression changes in ZnT genes. Data were obtained from triplicate experiments, and the expression of *CpTUB4* in YPD was used as the internal control for Δ/Δ Ct threshold cycle (CT) analysis. For statistical analysis, the Mann–Whitney test was used, *p < 0.05; **p < 0.01; ***p < 0.001; ^#^*p* < 0.0001.

**Figure 4.**
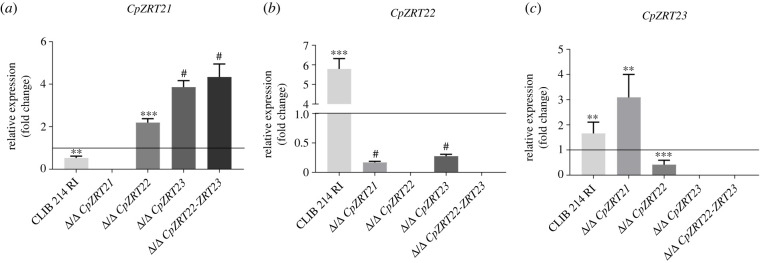
Acidic pH and low zinc concentration-dependent expression changes of *CpZRT2.* (*a–c*) Gene expressions represent the relative value to the expression of *CpTUB4* in acidic low zinc environment by Δ/Ct. Data show the means of triplicate experiments. For statistical analysis, Mann–Whitney test was used, *p < 0.05; **p < 0.01; ***p < 0.001; ^#^*p* < 0.0001.

Strains were cultured in YPD+ 100 unit ml^−1^ penicillin-streptomycin at 30°C with shaking (200 rpm) overnight. Then, 4 × 10^5^ cells were inoculated in the indicated media and cells were grown for 24 h. Cell harvesting and extraction was performed according to the manufacturer's protocols with the RiboPure RNA Purification Kit. Five hundred nanograms of RNA was transcribed by reverse transcription using the RevertAid first-strand cDNA synthesis kit protocol. Real-time PCR was performed by primers listed in electronic supplementary material, table S2 and the conditions were as follows: one cycle of denaturation for 3 min at 95°C; denaturation at 95°C for 10 s; 49 cycles of annealing at 60°C for 30 s, and elongation at 65°C for 30 s; with a final extension step at 72°C for 30 s. In the case of the determination of the predicted ZnT gene expressions in CLIB 214 wild-type strain Δ/Δ Ct threshold cycle (CT) analysis used, fold changes calculated from the normalized Cq values to the expression of *CpTUB4* housekeeping gene in YPD first in case of each condition and then to each gene (figure 2). The fold changes of ZRT2 orthologues were calculated by Δ/Ct analysis, the ZnT gene Cq values were normalized to the values of *CpTUB4* in acidic LZM medium in the case of each strain (figure 4).

### 2.6. Fluorescence staining

For intracellular zinc visualization, the Zinquin fluorescent probe was used. Overnight cultured yeast cells were inoculated in 1 x PBS, stained with 25 µM Zinquin and incubated for 40 min protected from light. The cells were then washed two times in 1 x PBS and prepared for flow cytometry or microscopy. Zinquin+ population was determined by subtracting the autofluorescence signal of the unstained cells using the gating strategy shown in electronic supplementary material, figure S5A.

To visualize the location of the GFP-tagged CpZrc1, the fungal cell wall, nucleus and vacuole were stained. Concanavalin-TRITC (final cc: 100 µg ml^−1^) or CalcoFluor White (final cc: 5 µg ml^−1^) were used for cell wall staining, the nucleus was labelled with DAPI (final cc: 50 µg ml^−1^), and the fungal vacuoles were stained with FM4-64 (final cc: 10 µg ml^−1^) in 200 µl final staining volume for 40 min at 30°C with shaking (200 r.p.m.) and protection from light. Afterwards, cells were washed in 1 x PBS two times and prepared for microscopic analysis.

### 2.7. Maintenance of J774.2 murine macrophages

J774.2 murine macrophages were cultured in Dulbecco's Modified Eagle Medium (DMEM, Lonza) supplemented with 100 unit ml^−1^ penicillin-streptomycin and 10 V/V % fetal bovine serum and incubated at 37°C, with 5% CO_2_. The medium was changed on confluent cultures every 3 days and immediately prior to the experiments.

### 2.8. Phagocytosis assay

J774.2 macrophages (1 × 10^6^) in 24-well plates were infected with the GFP-tagged strain or Alexa Fluor 647 (Invitrogen) stained (according to the protocol provided by Invitrogen) mutant strains in a 5 : 1 ratio (yeast cell: macrophage). After 0.5, 1, 2 and 3 h of coincubation, wells were washed with 1 x PBS, and then macrophages were collected. Macrophage singlet cells (10^4^) were examined with flow cytometry analysis (Amnis) per strain. Phagocytosis + macrophage population was determined by subtracting the autofluorescence signal of the uninfected J774.2 control (electronic supplementary material, figure S5B).

### 2.9. Fungal elimination assays

The killing efficiency of *C. parapsilosis* strains by J774.2 macrophages after 3 h of coincubation was determined by two approaches using an MOI of 5 : 1.

For the flow cytometry-based killing assay, 10^6^ J774.2 were challenged with 5 × 10^6^ yeasts/well in 24-well plates. Control wells contained yeast cells only. After 3 h of incubation, cells were collected and washed two times with FUN1 buffer (10 mM NaHEPES, 2 m/V % glucose), and then macrophage disruption was achieved by passage of the collected cells through insulin syringes. In order to separate yeast cells and macrophages debris, the fungal cell wall specific Calcofluor White was used together with the FUN1 dye to detect live fungal cells. The combined staining was set as follows: 195 µl of cell suspension (approx. 5 × 10^6^ cells) in FUN1 buffer + 1 µl FUN1 (100 µM) + 4 µl CalcoFluor White (2.5 mg ml^−1^). After 40 min of staining at room temperature (in the dark), cells were washed two times with FUN1 buffer, and live cells were enumerated by flow cytometry. Control wells contained only fungal cells in DMEM to determine 100% of live yeast cells. CalcoFluor White+ and FUN1 + populations were determined using the gating strategy added in electronic supplementary material, figure S5C.

A CFU-based fungal elimination assay was performed according to a standardized protocol with minor changes [29]. J774.2 cells (2 × 10^5^) were infected with 10^6^ yeast cells in 96-wells plates. Control wells contained only *Candida* cells in a similar amount. Three hours of post-infection, cells were collected and forced through an insulin needle. Suspensions was plated on YPD+1% penicillin-streptomycin plates and incubated at 30°C for 3 days. Killing efficiency by macrophages was determined as previously described by Németh *et al.* [29].

### 2.10. *Galleria mellonella* infection

*Galleria mellonella* survival experiments were performed as described previously [29]. The larvae were infected with 10 µl of *Candida* suspension containing 6 × 10^6^ yeast cells in 1 x PBS via their pro-legs using a 26 G needle. A group of larvae was inoculated with PBS as control, another group remained untreated. Larvae were incubated at 25°C, survival was monitored daily for 14 days.

### 2.11. Statistical analysis

Statistical significance was determined by unpaired *t*-tests or Mann–Whitney tests using the GraphPad Prism v. 7.0 software. The non-parametric Mann–Whitney tests routinely used to improve the analysis of the gene expression data to detect the differences between the median value of the groups with more power and strict approach than the other tests [30]. Differences between groups were considered statistically significant at *p* < 0.05.

## 3. Results

### 3.1. Identification of potential zinc transporters in *Candida parapsilosis*

**Figure 1.**
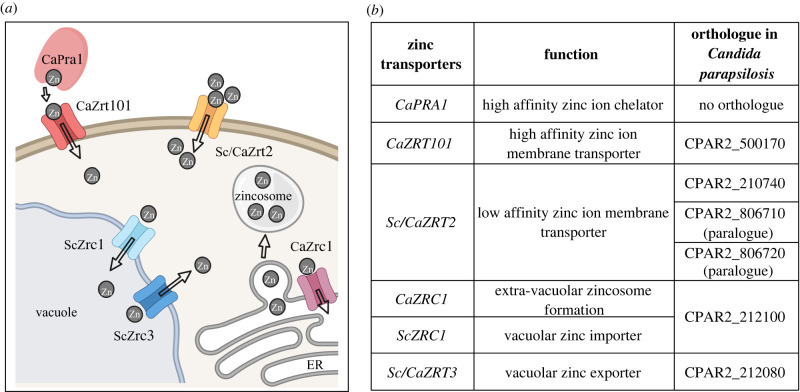
*In silico* ZnT analysis. (*a*) ZnTs in *S. cerevisiae* and *C. albicans*. (*b*) The target genes selected for KO generation in *C. parapsilosis.* Image (*a*) created with BioRender.com.

We performed *in silico* analyses to identify ZnTs in *C. parapsilosis* (figure 1). BLASTp analysis using *C. albicans* Zrt101, Zrt2, Zrt3 and Zrc1 as query identified six orthologues of known *C. albicans* and *S. cerevisiae*-ZnTs by nucleotide similarity (figure 1*b*). Notably, the orthologue of the CaPra1 zincophore zinc-binding protein was not identified in the genome of *C. parapsilosis* [12]. On the other hand, several putative zinc ion membrane transporters were detected. CPAR2_500170 was identified as the orthologous gene of the *CaZRT101*, which, in *C. albicans*, acts in conjunction with Pra1. We identified three transporters with high sequence similarity to *Sc/CaZRT2*; these were CPAR2_210740, CPAR2_806710 and CPAR2_806720, which we named *ZRT21*, *ZRT22* and *ZRT23*, respectively. Of these, CPAR2_210740 exhibited the highest sequence similarity to *C. albicans* zinc importer Zrt2. CPAR2_806710 and CPAR2_806720 appeared to be the result of an ancestral *ZRT2* gene duplication followed by recent tandem duplication in *C. parapsilosis*. CPAR2_212100 was identified as the orthologue of *CaZRC1* and *ScZRC1*, which function in zincosome and vacuolar zinc import, respectively. Both previously investigated Zrc1 transporters play important roles in zinc detoxification in these fungi. We also identified the orthologue of the vacuolar zinc exporter, *Sc/CaZRT3*, as CPAR2_212080 in the *C. parapsilosis* genome.

### 3.2. Zinc concentration-dependent expression of zinc transporters

Next, we aimed to investigate the expression patterns of the identified ZnT genes. We first determined low (0.05 mM), medium (1 mM) and high (10 mM) levels of zinc that could support the growth of *C. parapsilosis* CLIB 214 strain in LZM, pH 7.5 basal medium (figure 2*a*) and pH 4 (figure 2*e*). In order to compare the expression of genes under different zinc regimes, the expression of each transporter gene was analysed by Δ/Δ Ct where the internal control was the expression of *CpTUB4* in the wild-type strain in YPD medium. *CpZRT101* was expressed at its highest levels in low zinc media at neutral pH and progressively downregulated by the addition of zinc (figure 2*b–d*). At acidic pH, *ZRT101* was expressed at much lower levels, but again downregulated by the addition of exogenous zinc. Surprisingly, *CpZRT21* was transcribed at far lower levels, and with a higher expression at neutral pH than acidic. *CpZRT22* showed the highest transcription at low zinc and low pH while its paralogue, *CpZRT23*, exhibited the highest expression at medium zinc levels at neutral pH and was downregulated at acidic pH. We also measured *ZRC1* and *ZRT3,* which encode intracellular organellar zinc importers and exporters, respectively. At neutral pH *ZRC1* was modestly upregulated in response to increasing zinc, as might be expected for a gene involved in zinc detoxification. Finally, *ZRT3*, which encodes a vacuolar zinc exporter in other species, did not exhibit considerable differential regulation, except at low zinc and acidic pH, under which it was induced.

### 3.3. Generation of knock-out mutants and their phenotypical characterizations

To study the potential role of the identified ZnTs in *C. parapsilosis* zinc homeostasis, we generated homozygous deletion mutants of each of the six genes identified in figure 1. Two independent null mutants were created per gene, using the auxotrophy complementation method developed by Holland *et al*. [25]. The two independent mutants of each homozygous KO strains showed the same phenotype in all cases. To generate reintegrant strains, *CpZRT21* and *CpZRC1* were introduced into the *CpNEUT5 L* neutral locus of each null mutant strain. The reintegration of a single copy of *CpZRC1* did not complement the phenotype of Δ/Δ *CpZRC1*, so *CpZRC1* was placed under control of the *CaTDH3* promoter in the *CpNEUT5 L* region.

### 3.4. Characterization of the zinc uptake system

#### 3.4.1. Zinc concentration and pH-dependent growth of Δ/Δ *CpZRT21*

**Figure 3.**
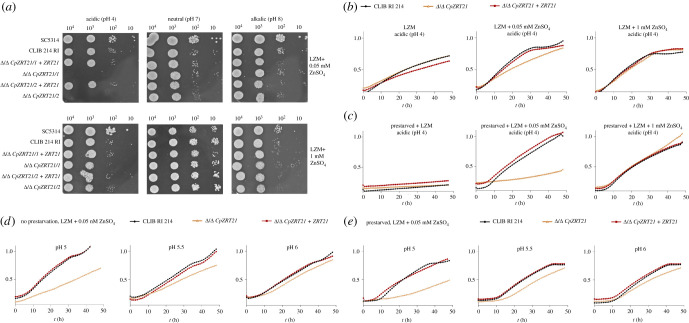
The enhanced growth of the *C. parapsilosis* Δ/Δ *CpZRT21* mutant upon alkalinization. (*a*) Spot assays were performed with the *CpZRT21* null mutants on acidic (pH = 4), neutral (pH = 7) and alkalic (pH = 8) solid media with different zinc concentrations. (*b*) Figures represent the growth kinetics of the indicated strains under acidic (pH4) environmental conditions in different zinc-supplemented media as indicated. (*c*) For zinc pre-starvation, strains were pre-cultured in liquid YPD medium, and then inoculated into a liquid LZM medium for 24 h. Cells were washed with 1 mM EDTA before the growth test. (*d*) Growth assays in discrete pH valued (pH 5–pH 6) LZM medium supplemented with 0.05 mM zinc without zinc pre-starvation and (*e*) with LZM pre-starvation. The spot assays were performed three times. Growth curves represent mean data of three independent experiments performed in triplicates.

Based on our phylogenetic analysis of zinc importers in *C. parapsilosis* (figure 1), the most striking feature of this species was the gene expansion of *ZRT2*. This transporter is critical for zinc uptake and growth of *C. albicans* in acidic environments. We therefore sought to functionally characterize the orthologue of Zrt2 in *C. parapsilosis* (which we have termed Zrt21), as well as its paralogues—Zrt22 and Zrt23. Independent Δ/Δ *CpZRT21*, Δ/Δ *CpZRT22* and Δ/Δ *CpZRT23* strains, as well as a double mutant lacking both *ZRT22* and *ZRT23*, were generated and assayed for growth at acidic, neutral and alkaline environments. Only deletion of *ZRT21* rendered *C. parapsilosis* unable to grow on low zinc agar plates at acidic pH (figure 3*a*). In liquid media, wild-type, Δ/Δ *CpZRT21* and Δ/Δ *CpZRT21+ CpZRT21* grew relatively poorly in unsupplemented LZM and growth was enhanced by the addition of zinc, with the Δ/Δ *CpZRT21* mutant exhibiting a moderate growth defect in comparison to wild-type and revertant in LZM + 0.05 mM zinc. We therefore extended this analysis by first preculturing cells under zinc limitation to deplete intracellular zinc reserves before the inoculation of growth assays. Figure 3*c* shows that when pre-starved of zinc, all strains failed to grow upon subsequent inoculation of LZM without zinc. Comparing the growth of normal- versus zinc pre-starved cells in unsupplemented LZM suggests that *C. parapsilosis* may be using intracellular zinc stores. When zinc pre-starved cells were used to inoculate LZM + 0.05 mM zinc, the Δ/Δ *CpZRT21* mutant exhibited a severe growth defect in comparison to wild-type and revertant. This suggests that, like its *C. albicans* orthologue Zrt2, Zrt21 plays a vital role in zinc uptake in acidic media (figure 3*a*,*c*), while the transporter is dispensable for growth at neutral–alkaline pH (figure 3*a*). We therefore performed growth analysis at discrete acidic pH values supplemented with 0.5 mM zinc either or without zinc pre-starvation to probe the granularity of Zrt21-dependent growth. Figure 3*d,e* shows that Δ/Δ *CpZRT21* growth was still strongly reduced at pH 5.0, had a more moderate growth defect at pH 5.5, and grew at close to wild-type rates at pH 6.0. These data show that, as pH increases towards neutrality, Zrt21 becomes redundant.

#### 3.4.2. Expression compensation of *CpZRT2* genes

Because of the complexity of the zinc uptake repertoire of *C. parapsilosis*, and the essentiality of *ZRT21* in acidic environments, we next assessed the effect of *ZRT21*, *ZRT22* and *ZRT23* deletion on the expression of these genes. For this analysis, we selected liquid LZM pH 4, supplemented with 0.05 mM zinc, inoculated with YPD pre-grown cells as this condition permitted the growth of all strains (figure 3) and in which we inferred the existence of Zrt21-dependent zinc uptake from the surrounding media (compare Δ/Δ *CpZRT21* figure 3*b* versus figure 3*c*, central panels). The deletion of the *CpZRT22* and *CpZRT23* (and also the double disruption of the genes) resulted in an increase in the expression of *CpZRT21* (figure 4*a*). In addition, the absence of *CpZRT21* repressed the expression of *CpZRT22* but increased the transcript level of *CpZRT23*. Finally, the expression of *CpZRT22* and *CpZRT23* decreased in the Δ/Δ *CpZRT23* and Δ/Δ *CpZRT22* mutants, respectively (figure 4*b*,*c*). These data point towards a complex regulatory interplay among these zinc import genes in *C. parapsilosis*.

### 3.5. Functional characterization of *CpZRC1*

#### 3.5.1. *CpZRC1* is essential for zinc detoxification

**Figure 5.**
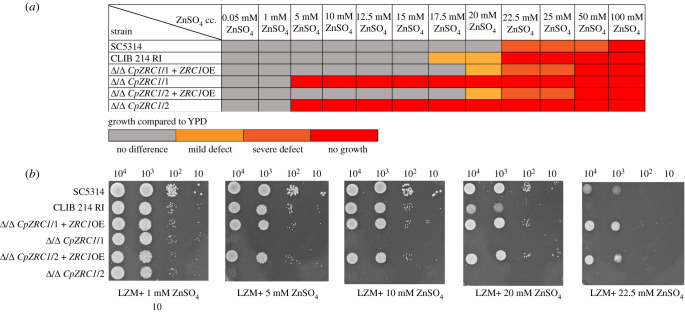
Viability of the generated mutant strains in the presence of different zinc concentrations. The homozygous deletion mutants were applied for spot assays and their growth was examined in the presence of 31 different stress conditions. The viability of *CpZRC1* deletion mutants altered significantly in high zinc concentrations. (*a*) Summary of the results of all performed experiments. (*b*) Representative images of spot assays were performed with the *ZRC1* strains.

**Figure 6.**
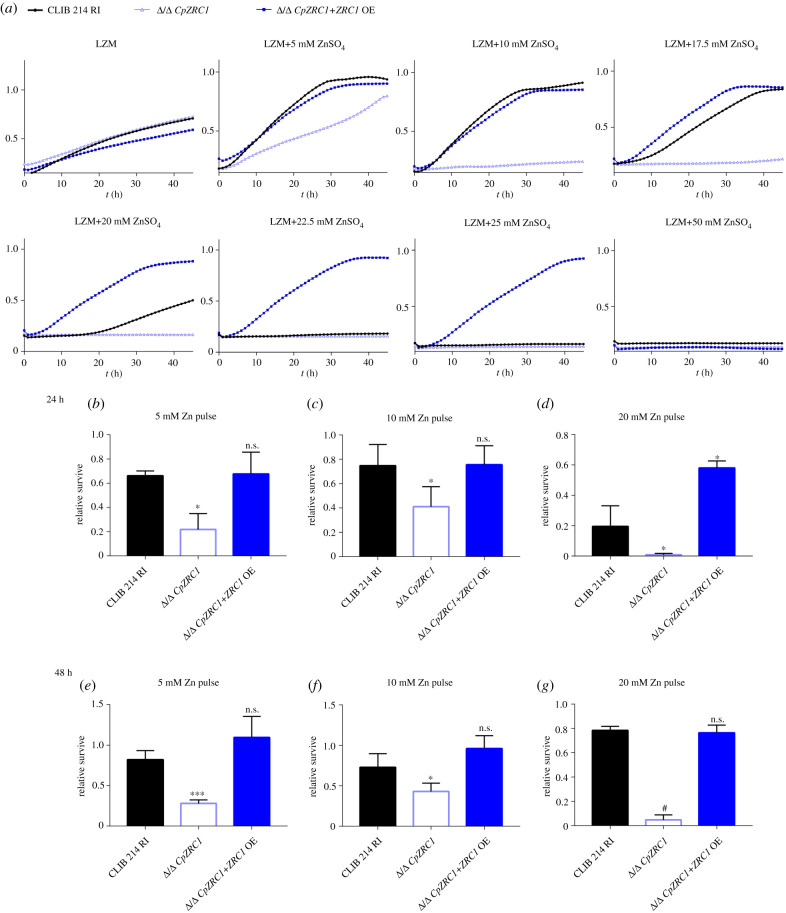
*CpZRC1* is essential for zinc detoxification. (*a*) Growth kinetics of Δ/Δ *CpZRC1* strains in different zinc-containing liquid media. (*b–g*) Results of zinc survival assays. After 5, 10 and 20 mM zinc pulses for 24 and 48 h, 500 cells were plated on YPD then viable cells were counted. The growth curves represent the results of three independent experiments performed in triplicates. Relative survival was determined from three independent experiments performed in triplicates. For statistical analysis, unpaired *t*-tests were used, *p < 0.05; **p < 0.01; ***p < 0.001; ^#^*p* < 0.0001.

We first characterized zinc tolerance in *C. parapsilosis* in comparison to *C. albicans*. Figure 5 shows that when cultured on LZM agar plates, *C. parapsilosis* wild-type exhibited robust growth with up to 10 mM zinc supplementation. At 20 mM zinc, growth of *C. parapsilosis* but not *C. albicans* was inhibited, and at greater than or equal to 22.5 mM zinc, colony formation of *C. parapsilosis* was blocked completely. The deletion of *ZRC1* had no impact on *C. parapsilosis* on LZM media containing 1 mM zinc; however, at 5 mM and above, no growth was observed for the Δ/Δ *CpZRC1* mutant. Similar results were observed in liquid media: *C. parapsilosis* wild-type tolerated up to 17.5 mM zinc in liquid LZM media, with growth substantially reduced at 20 mM and above whereas the growth of the Δ/Δ *CpZRC1* mutant was inhibited at 5 mM and above (figure 6). Again, *TDH3* promoter-driven overexpression of *ZRC1* permitted robust fungal growth at zinc levels well beyond that tolerated by wild-type *C. parapsilosis* (figures 5 and 6).

Besides reduced growth (figures 5 and 6), the viability of Δ/Δ *CpZRC1* cells also decreased at greater than or equal to 5 mM ZnSO_4_ concentrations, as reflected by the significantly lower number of colonies on YPD plates recovered following 5, 10 and 20 mM zinc pulses for both 24 and 48 h (figure 6*b–g*). Notably, the overexpression of *CpZRC1* overcame this affect both on solid plates and in liquid medium. Interestingly, both the growth and survival of the *Δ*/*Δ CpZRC1* +*ZRC1*OE strain exceeded that of the wild-type strain's, both on solid plates and in liquid medium supplemented with higher amounts of zinc (20, 22,5, 25 mM ZnSO_4_) (figures 5 and 6).

#### 3.5.2. Zincosome formation in *Candida parapsilosis*

**Figure 7.**
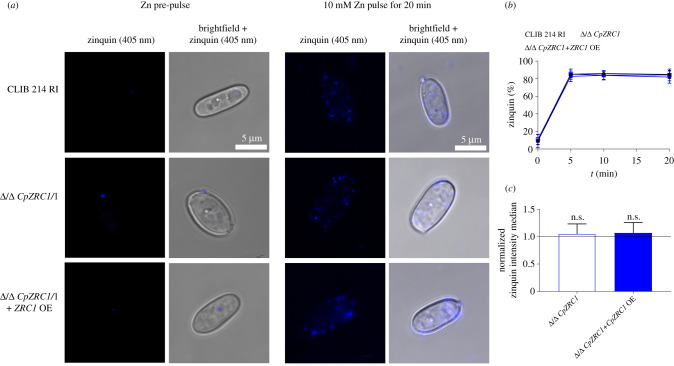
Zincosome formation in *C. parapsilosis* is CpZrc1 independent. Strains were cultured in liquid YPD medium, then inoculated into LZM liquid medium supplemented with high level (10 mM) of zinc. Zinquin fluorescent dye was used to visualize intracellular zincosomes. (*a*) Images represent the localization of zincosomes after 20 min of 10 mM zinc pulse. (*b*) Percentage of Zinquin+ cells at indicated timepoints. (*c*) Figure represents the intensity median of Zinquin in cells after 20 min by flow cytometer. Experiments were performed three times. For statistical analysis, unpaired *t*-tests were used, *p* < 0.05.

Results of the phenotypic analyses suggested that *CpZRC1* is essential for zinc detoxification in *C. parapsilosis*. *Saccharomyces cerevisiae* Zrc1 is a vacuolar zinc importer, and *C. albicans* Zrc1 is responsible for zincosome (intracellular zinc store) formation [11,13]. Thus, we aimed to examine whether Zrc1 has a similar function in *C. parapsilosis*. For this purpose, we used Zinquin, a dye that is used to stain zincosome zinc. *Candida parapsilosis* strains were challenged with a 10 mM zinc for 5, 10 and 20 min. After zinc treatment, the intracellular localization of Zinquin was determined via confocal microscopy (figure 7*a*). In addition, the percentage of Zinquin+ population by flow cytometry (figure 7*b*) and the median of Zinquin signal intensity (figure 7*c*) by flow cytometry. The wild-type *C. parapsilosis*, Δ/Δ *CpZRC1*, Δ/Δ *CpZRC1* + *ZRC1*OE strains were able to form zincosomes equally in a rapid way following stimulation with 10 mM of zinc treatment after 5–20 min.

**Figure 8.**
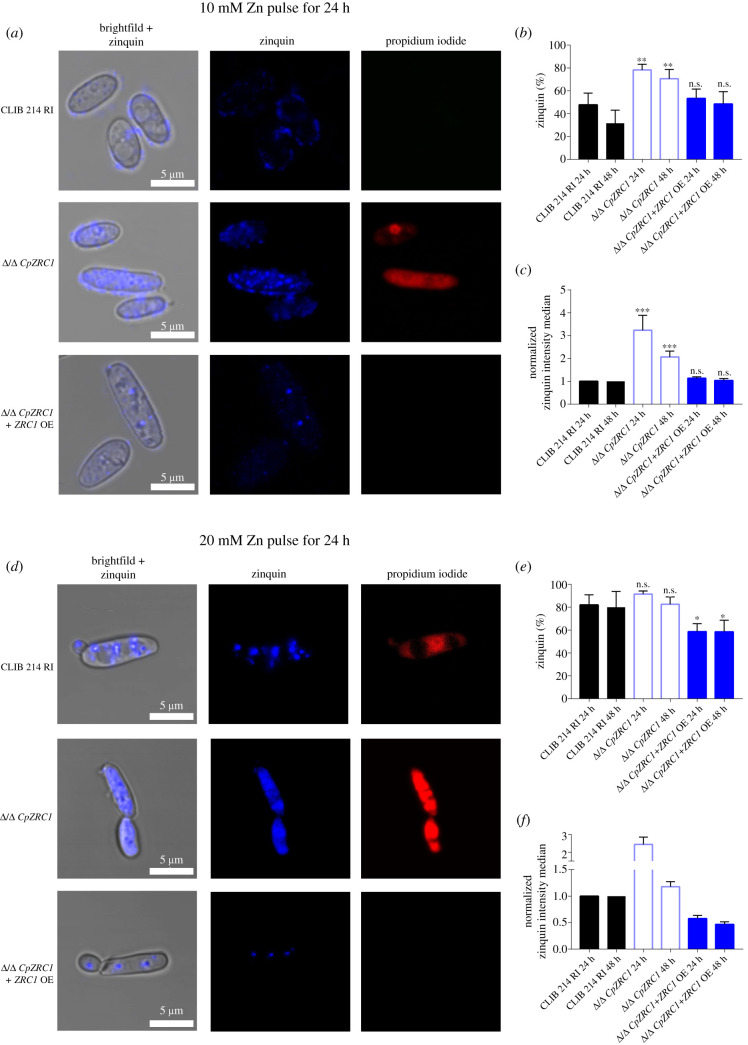
Zincosome formation under toxic zinc levels. Strains were grown in liquid YPD medium and then inoculated into LZM liquid medium supplemented with 10 mM or 20 mM zinc. Cells were then stained with Zinquin (zincosomes) and Propidium Iodide (viability). (*a,d*) Images represent *CpZRC1* strains challenged with 10 mM and 20 mM zinc for 24 h. (*b,e*) The Zinquin+ population was determined after zinc treatment. (*c,f*) Zinquin intensity median presented after the zinc pulse. Experiments were performed three times using flow cytometer. For statistical analysis, unpaired *t*-tests were used, *p < 0.05; **p < 0.01; ***p < 0.001.

Next, the effect of prolonged zinc treatment was tested. Cells were treated with 10 or 20 mM zinc for 24 h followed by Zinquin staining and propidium iodide to assess cell viability (figure 8*a*,*d*). Interestingly, the Δ/Δ *CpZRC1* strain exhibited more intense Zinquin staining than wild-type and revertant cells. This contrasts with *C. albicans* Δ/Δ *CpZRC1* cells, which exhibit reduced zincosomes and Zinquin staining [13]. In the absence of *CpZRC1*, the 10 mM concentration of zinc pulse for 24 and 48 h increased the Zinquin+ population and Zinquin signal intensity but also reduced viability (figure 8*a–c,e,f*). In line with our previous observations, the 20 mM zinc pulse proved to be toxic for wild-type cells, while the Δ/Δ *CpZRC1* + *ZRC1*OE strain was able to survive (figure 8*d*). Furthermore, in the wild-type and *CpZRC1* mutant strain, the Zinquin+ population increased and the zincosomes appeared to leak resulting in homogeneous Zinquin signals (figure 8*d,e*). The reintegrant Δ/Δ *CpZRC1* + *ZRC1*OE strain showed a phenotype similar to that of the wild-type only in the case of 10 mM zinc treatment.

#### 3.5.3. Intracellular localization of CpZrc1

**Figure 9.**
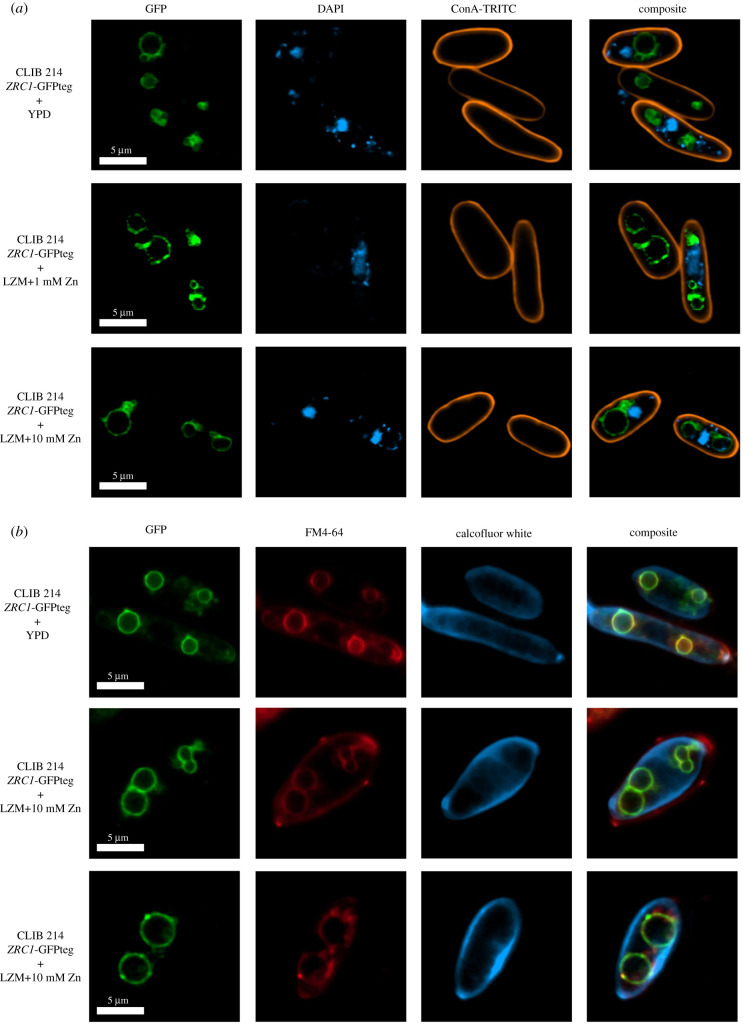
CpZrc1 co-localizes to vacuolar membrane. The CLIB 214 *ZRC1*-GFP-tagged strains were grown in liquid YPD medium and then inoculated in liquid YPD or LZM medium containing 1 mM/ 10 mM zinc. Cells were incubated for 24 h. (*a*) Then, cells were co-stained with ConcanavalinA-TRITC (cell wall) and DAPI (nucleus). (*b*) Cells also stained with FM4-64 (vacuole) and CalcoFluor White (cell wall) dye. Representative pictures of the intracellular CpZrc1 localization were taken by a confocal microscope. The experiment was performed twice.

To visualize the localization of CpZrc1, we tagged the C-terminus of *CpZRC1* with GFP. The generated strain was inoculated in YPD or LZM liquid medium supplemented with 1 mM (medium level) or 10 mM (high level) of zinc and incubated for 24 h. After incubation, ConcanavalinA-TRITC (orange) and DAPI (blue) were applied as cell wall and nucleus staining (figure 9*a*). In addition, cell wall dye CalcoFluor White (blue) and vacuole stain FM4-64 (red) were also used to characterize CpZrc1 localization (figure 9*b*). Confocal microscopy revealed that CpZrc1 localizes outside of the nucleus, was absent from the cell wall, and instead co-localizes with the FM4-64 signal in the vacuolar membrane. The concentration of zinc supplementation did not affect the signal of GFP-tagged CpZrc1.

### 3.6. Role of *CpZRT21* and *CpZRC1* in virulence

#### 3.6.1. Phagocytosis is not affected by *CpZRT21* and *CpZRC1*

**Figure 10.**
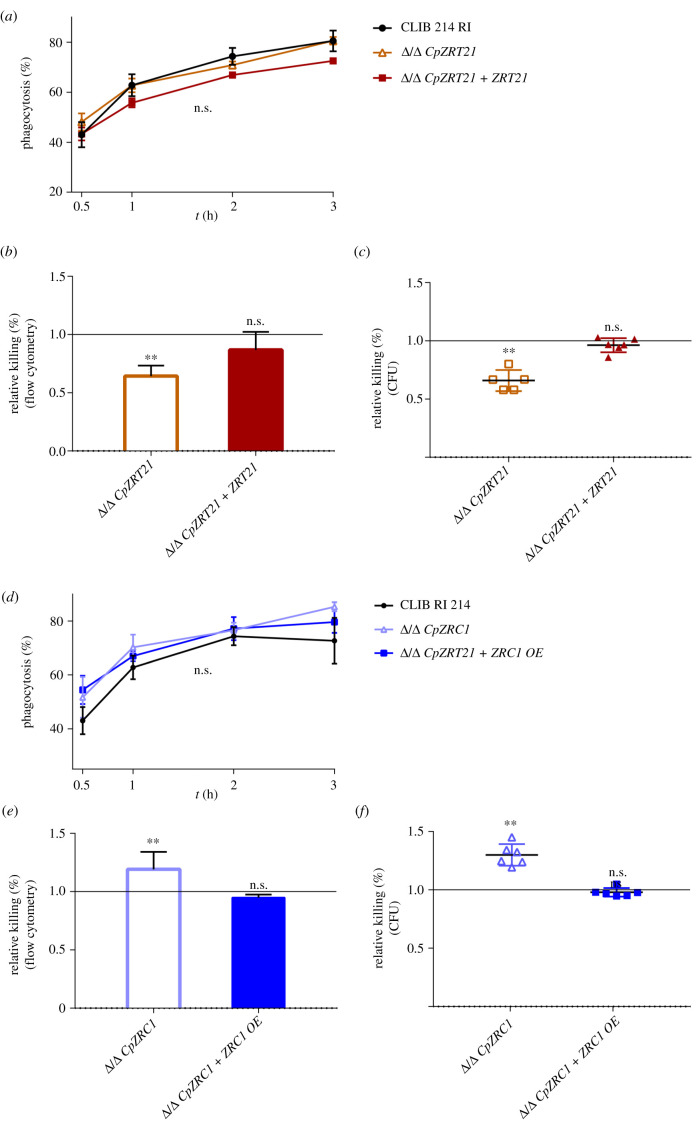
The absence of *CpZRT21* increases *C. parapsilosis* susceptibility to killing whereas *CpZrc1* protects against elimination by J774.2 macrophages. (*a,d*) J774.2 murine macrophages and the GFP-tagged or AlexaFluor647-stained *C. parapsilosis* strains were co-incubated for the indicated time points, and then phagocytosis kinetics was determined by flow cytometry. (*b,e*) The killing efficiency of J774.2 macrophages was determined after 3 h. Calcofluor White and the two-colour fluorescent FUN1 were applied as a co-staining method for determining yeast cell viability. (*c,f*) shows the results of killing assay performed by CFU counting method from YPD plates. The MOI = 1 : 5 was used for the experiments. Data were normalized to the elimination rate of the wild-type. Experiments were performed at least three times. For statistical analysis, unpaired *t*-tests were used, *p < 0.05; **p < 0.01.

Macrophages play key roles in controlling pathogenic fungi. Interestingly, these phagocytes can mount either zinc-starvation or intoxification responses against phagocytosed fungi [7,31]. We therefore assessed the interaction between *C. parapsilosis* ZnT mutant strains and J774.2 murine macrophages. We first examined phagocytosis efficiency 0.5, 1-, 2- and 3-hours of post-infection using flow cytometry analysis. Each strain was labelled with Alexafluor 647 succinimidyl ester in order to determine the phagocytosis+ macrophage population after the indicated time points. We found no significant difference between the uptake of *ZRT21*, *ZRC1* mutants and the wild-type strain regardless of the selected time points (figure 10*a*,*d*).

#### 3.6.2. *CpZRT21* and *CpZRC1* influence fungal killing by murine macrophages

Fungal cell elimination efficiency by J774.2 macrophages was determined 3 h post-infection by flow cytometry analysis, using FUN1 and Calcofluor White staining. Killing efficiency was also measured by CFU counting of recovered yeast cells 3 h after yeast-J774.2 interactions. The results of both methods revealed that deletion of *CpZRT21* led to less efficient fungi elimination (figure 10*b,c*). By contrast, the lack of *CpZRC1* led to more efficient killing by macrophages (figure 10*e*,*f*). The reintegrant strains showed a phenotype similar to that of the wild-type. Because Zrc1 is essential for zinc detoxification, these data suggest that *C. parapsilosis* may experience zinc toxicity within J774.2 macrophages.

#### 3.6.3. *CpZRT21* and *CpZRC1* is not essential for *in vivo* fungal colonization of *Galleria mellonella*

**Figure 11.**
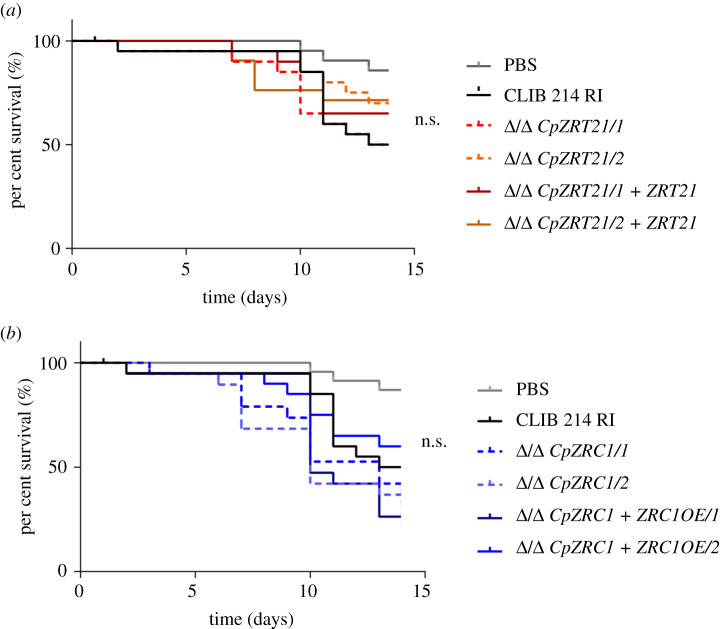
In *vivo Galleria mellonella* infection with wild-type, *CpZRT21* and *CpZRC1* mutant strains. After 7 days of acclimatization, *Galleria mellonella* larvae were infected with the indicated fungal strains. The surviving capability of the larvae was determined in the next 14 days. Twenty larvae were used per strain. For statistical analysis, the Mantel–Cox test was used, *p < 0.05; **p < 0.01; ***p < 0.001.

Next, we investigate the role of the described ZnTs of *C. parapsilosis* in the *Galleria mellonella* insect infection model. Larvae were infected with the wild-type, Δ/Δ *CpZRT21* and Δ/Δ *CpZRC1* strains then the survival rate was monitored for 14 days. We found no significant difference in survival of the larvae between the mutant strains and wild-type in this model (figure 11).

## 4. Discussion

Zinc is essential for cellular life and its uptake by fungal pathogens is crucial for maintaining both viability and pathogenicity [3]. In this study, we aimed to assess the role of zinc uptake and homeostasis in the human fungal pathogen *C. parapsilosis* and to examine their role in virulence. Based on *in silico* predictions, transporters for zinc uptake (Sc/CaZrt2, CaZrt101), detoxification (Sc/CaZrc1) and vacuolar export (ScZrt3) were identified in *C. parapsilosis*, similarly to *C. albicans* and *S. cerevisiae*. However, we did not find a potentially functional CaPra1 orthologue in the *C. parapsilosis* genome.

The availability of zinc is low within the host due to nutritional immunity. In fungal pathogens, zinc uptake required for survival and also host colonization is mediated by Zip transporter families [4]. Our *in silico* analyses predicted four such cellular zinc importer related to *C. albicans* Zrt101 and Zrt2. CpZrt101 showed similarity with the CaZrt101 membrane-localized ZnT and its transcriptional regulation was similar to that of *CaZRT101*, exhibiting increased expression at neutral than acidic pH. This is interesting because the orthologue of *Candida ZRT101* in the distantly related mould *Aspergillus fumigatus zrfC* is also positively regulated by neutral pH. This indicates that pH-dependent regulation of this ZnT may be conserved across the ascomycota phylum of fungi. In this context, it is worthwhile noting that although Zrt101 is missing from the model yeast *S. cerevisiae*, orthologues are present in most ascomycete fungi [12].

The orthologue of the Zrt101-associated zincophore, the CaPra1 zincophore, was not identified in the genome of *C. parapsilosis* as previously reported [12]. By contrast, three genes (*CpZRT21*, *CpZRT22* and *CpZRT23*) showed high similarity with the acid-adapted transporter Sc/Ca*ZRT2*. *CpZRT22* and *CpZRT23* appear to be the result of an ancestral duplication of the conserved *CaZRT2/CpZRT21*, followed by a subsequent tandem duplication [32]. *PRA1* loss and *ZRT2* expansion in *C. parapsilosis* is noteworthy from ecological perspective. This yeast is a common colonizer of human skin which is mildly acidic. Because Pra1 is adapted for zinc uptake at neutral–alkaline pH and Zrt2 at acidic, the genetic arrangements observed in *C. parapsilosis* may be associated with such pH adaptation.

Our gene expression analysis revealed that *CpZRT21* was transcribed at lower levels at acidic versus neutral pH. This is in contrast with the *ZRT21* orthologue in *C. albicans* (*ZRT2*) that is more highly expressed at acidic than neutral–alkaline pH [14,33]. Nevertheless, *CpZRT21* did exhibit downregulation upon zinc supplementation at both pH values, as expected for a gene involved in zinc acquisition, and as previously observed for *CaZRT2*.

Notably, all four of these previously described yeast species encode only two Zip-type plasma membrane zinc importers each. *CpZRT22* exhibited the expression profile of a canonical acid-adapted zinc uptake with increased transcripts level at depleted zinc and low pH values. Its paralogue gene *CpZRT23* showed the highest expression at neutral pH, and it was downregulated significantly by lowering the pH in the medium. Interestingly, in acidic media, *CpZRT23* was more highly expressed at medium (1 mM) zinc than at either low (0.05 mM) or high (10 mM) zinc. This bimodal pattern in response to zinc is reminiscent of the low-affinity zinc importer *ZRT2* from *S. cerevisiae* [34]. All of the Zrt2-like genes' transcript levels altered in response to zinc.

Together, these phylogenetic and gene expression observations indicate that the zinc import machinery of *C. parapsilosis* is more complex than that of the other studied yeast species.

The previously investigated yeast species *S. cerevisiae*, *C. gattii C. albicans* and *C. dubliniensis* each encode only two Zip-type cellular zinc importers. In *S. cerevisiae*, *C. gattii* and *C. dubliniensis*, the Zap1 transcription factor is responsible for the regulation of Zip importers in response to environmental zinc levels [25–27]. *Candida albicans* Zap1 also regulates the zinc regulon in this species [35]. Thus, a similar regulator to Zap1 might also be present in *C. parapsilosis*. Indeed, a BLASTp search using *C. albicans* Zap1 as query identified a predicted transcription factor with high-sequence similarity (1 × 10^−176^) in *C. parapsilosis* called CPAR2_403080.

In liquid media, *C. parapsilosis* grew poorly under zinc limitation and growth was restored upon zinc supplementation. Δ/Δ *CpZRT21* exhibited only a minor growth defect compared to wild-type and revertant in LZM + 0.05 mM zinc. The pre-culture for these growth assays was YPD, which contains approximately 29 µM zinc [36]. We reasoned that the fungus may have stored some of the zinc from this pre-culture as has been previously shown for *S. cerevisiae* [11]. To test this hypothesis, cells were first pre-cultured under zinc-limiting conditions before inoculation. We found that cells from a zinc-depleted pre-culture were unable to grow when inoculated into LZM (figure 3). This indicates that *C. parapsilosis*, like *S. cerevisiae*, may have the capacity to generate and use intracellular stores (left-hand panel of figure 3*b,c).* When cells from a zinc-depleted pre-culture were used to inoculate LZM + 0.05 mM zinc, wild-type and Δ/Δ *CpZRT21* +*ZRT21* strains grew well, but Δ/Δ *CpZRT21* growth was severely inhibited. This suggests that Zrt21 is essential for zinc uptake and growth when the cells must secure low levels (0.05 mM) of zinc from the environment. Finally, supplementation with excess zinc (1 mM) bypassed the requirement for Zrt21.

Interestingly, of the four identified ZnTs, only *CpZRT21* showed a growth defect under zinc-depleted acidic environmental conditions. However, the transcription of the *CpZRT21, CpZRT22* and *CpZRT23* in this media revealed interesting patterns of expression. Compared to wild-type cells, the deletion of *ZRT22* and *ZRT23* either alone or in combination resulted in the upregulation of *ZRT21*. Such compensatory upregulation is common among genes in the same micronutrient uptake pathway. The deletion of *ZRT22* downregulated *ZRT23* while the absence of *ZRT23* downregulated *ZRT22*. These two genes are, however, located at the same genetic locus, and we cannot rule out some perturbation of regulation due to gene deletion itself. The most interesting observation was that the deletion of *ZRT21* (which is located on a different chromosome than *CpZRT22/23*) resulted in the simultaneous downregulation of *CpZRT22* and upregulation of *CpZRT23*. While it is possible that the zinc restriction experienced by *Δ*/*Δ CpZRT21* cells may contribute towards the observed differential regulation of *CpZRT22* and *CpZRT23*, we think this is unlikely as *CpZRT22* was progressively upregulated under low zinc conditions in acidic media whereas *CpZRT23* expression was only modestly higher. These results suggest that the Zrt2-based zinc uptake in *C. parapsilosis* regulated by *CpZRT21*, *CpZRT22* and *CpZRT23* might be under the same co-regulation process. It could explain why neither the single deletion nor the double deletion of *CpZRT22* and *CpZRT23* resulted in a loss of viability. Hence, CpZrt21 may compensate for the lack of *ZRT22* and *ZRT23*. In the future, this hypothesis could be confirmed with the generation and thorough characterization of a Δ/Δ *CpZRT21/ZRT22/ZRT23* triple mutant strain.

In a host niche, microorganisms can encounter potentially toxic levels of zinc following phagocytosis by immune cells. Recent studies established that macrophages are able to harm pathogens via sequestration of a toxic amount of zinc ions in the phagolysosome to kill or limit their growth [6,7]. Notably, several pathogenic species possess strategies to detoxify zinc to survive such an antimicrobial activity, and this ability is considered to be an important virulence factor [6,13]. Based on the orthology relations with *S. cerevisiae* and *C. albicans,* we predicted the zinc detoxification transporter Zrc1 to be CPAR2_212100 in *C. parapsilosis*. It should be noted that all *ZRC1* orthologues studied to date [13,37–39] play important roles in zinc detoxification. At acidic pH, *CpZRC1* expression increased with decreasing zinc levels. While this may appear counterintuitive, it has been described for other fungi and may represent an adaptive mechanism to protect cells against future zinc shock. The fact that we observed this phenomenon at acidic, but not neutral/alkaline pH, may be due to the much greater solubility, and thus bioavailability, of zinc ions at lower pH [40]. We also found that deletion of *CpZRC1* resulted in a viability defect at 5 mM zinc-containing conditions, which also supports the zinc detoxification role of CpZrc1. Notably, the reintegration of one copy of *CpZRC1* with its native promoter did not complement the wild-type phenotype, which led us to overexpress *CpZRC1* using *CaTDH3* promoter in the Δ/Δ *CpZRC1* mutant strain. The overexpression of *CpZRC1* restored the wild-type phenotype at 5–10 mM zinc-containing environment. At greater than or equal to 17.5 mM zinc concentrations, *CpZRC1* overexpression even aided fungal survival. The zinc survival assay also supported the zinc detoxification role of CpZrc1: the deletion of *CpZRC1* resulted in growth deficiency after 24 and 48 h of 5 to 20 mM of zinc treatment. Zrc1 operates two different zinc detoxification processes in *S. cerevisiae* and *C. albicans*. In *S. cerevisiae*, ScZrc1 is responsible for vacuolar zinc import, while it defends against high amounts of intracellular zinc via CaZrc1-dependent zincosome formation in *C. albicans* [13,41]. To determine the function of Zrc1 in *C. parapsilosis*, a zincosome-specific *fluorescent* stain was used. The toxic (10 mM) amount of zinc treatment resulted in rapid zincosome formation in *C. parapsilosis* after 5–20 min of incubation, and the Δ/Δ *CpZRC1* mutant was also able to form the same amount of zincosomes as the wild-type. The 10 mM zinc treatment for 24 and 48 h was toxic for the Δ/Δ *CpZRC1* strain, which correlated with the high amount of zincosomes and dead cells detected under the same circumstances by confocal microscopy and flow cytometer. Interestingly, while the 20 mM zinc treatment (which is already toxic for the wild-type) resulted in high amount of zincosome formation in the wild-type and Δ/Δ *CpZRC1* strains, the Zinquin level in the Δ/Δ *CpZRC1* + *ZRC1*OE strain was reduced. In *C. parapsilosis*, zincosome formation appeared overwhelmed in the absence of CpZrc1, so zincosomes were overloaded with zinc ions in the presence of high levels of zinc. We hypothesized that CpZrc1 either has another zinc detoxification target organelle or (in contrast with *C. albicans*) it is localized on the membrane of vacuoles. Indeed, C-terminal tagging of Zrc1 with GFP and confocal microscopy with cell wall, nuclear and vacuolar co-staining revealed predominantly the vacuolar localization of CpZrc1. Therefore, despite being phylogenetically more closely related to *C. albicans*, *C. parapsilosis* Zrc1 subcellular localization more closely resembles that of *S. cerevisiae* and *C. neoformans* [38,41].

Acquisition of zinc in environments where the micronutrient is limited conditions and defence against toxic zinc concentrations are both important for pathogenic fungi to survive and successfully colonize a host. In order to investigate the interaction between the host and the ZnT mutants, J774.2 murine macrophages, *G. mellonella* larvae were used to assess the mutant strain's virulence properties both *in vitro* and *in vivo*. Phagocytosis of the Δ/Δ *CpZRT21* and Δ/Δ *CpZRC1* strains by the J774.2 cells was similar to that of the wild-type in all time points. However, Δ/Δ *CpZRC1* cells were cleared more effectively by the macrophages, while Δ/Δ *CpZRT21* cells were less effectively killed. Macrophages are able to increase phagolysosomal zinc to aid the clearance of pathogens. In the case of *M. tuberculosis,* zinc rapidly accumulates in the phagolysosomes [6]. A recent study also revealed a zinc-based killing process by murine bone marrow-derived macrophages during *C. glabrata* infection [7]. The behaviour of our zinc uptake and zinc detoxification defective mutants suggests that *C. parapsilosis* cells might trigger a similar effect once taken up by J774.2 macrophages. The Δ/Δ *CpZRT21* mutant may be partially defended by the excess zinc as it is lacking the major plasma membrane zinc importer. Simultaneously, increased killing of the Δ/Δ *CpZRC1* cells, which lack the vacuolar membrane-localized zinc detoxification system, indicate that *C. parapsilosis* may be experiencing zinc poisoning within these immune cells.

*Galleria mellonella* larvae can use as a model of humoral and cellular innate immune responses. According to Trevijano-Contador & Zaragoza, *Galleria* larvae are suitable for the investigation of innate immunity and its response to fungal infections [42]. *Galleria mellonella* were used to uncover the virulence properties of many mutant strains and also to detect the effect of nutrient deprivation on the sensitivity of the larvae for *C. albicans* infection [43,44] These above-mentioned results showed a strong correlation with mammalian *in vivo model* approaches, so we used this insect organism as a substitute for vertebrate *in vivo* experiments.

To uncover the effects of *CpZRT21* and *CpZRC1* deletion *in vivo,* we infected larvae with the indicated strains and monitored their survival for 14 days. During the *in vivo* experiments, we detected no virulence alterations among the generated mutant strains in a 14-day-long survival experiment using *G. mellonella* larvae. Overall, we can assume that the lack of CpZrt21 membrane ZnT and CpZrc1 vacuolar zinc importer do not affect the immune response and the survival rate of the healthy nutrient-supplied *Galleria mellonella* larvae to *C. parapsilosis* infection. Additionally, a more in-depth analysis is considered to uncover the response and the survival rate of the nutrient deprivation in *Galleria mellonella* during *Candida parapsilosis* infection.

**Figure 12.**
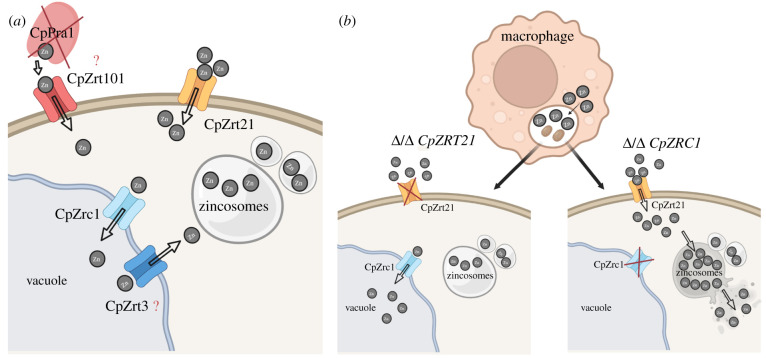
Summary of the zinc homeostasis in *C. parapsilosis*. (*a*) shows the described zinc uptake and zinc storing mechanisms of *C. parapsilosis*. (*b*) Effects of the hypothetical zinc poisoning-based *C. parapsilosis* elimination by macrophages in the absence of *CpZRT21* or *CpZRC1*. Images created with BioRender.com.

In summary, we have identified and characterized for the first time five ZnTs in *C. parapsilosis* (figure 12*a*). Similar to *C. albicans*, growth of *C. parapsilosis* in an acidic environment is mediated exclusively by the membrane ZnT CpZrt21. However, in this species, two additional Zrt2 orthologues are also present that may be under the same regulation as *CpZRT21* in the setting of zinc-limited conditions. We revealed that *C. parapsilosis* forms zincosomes under high zinc-supplemented environmental conditions, although, in contrast with *C. albicans,* in a Zrc1-independent way. Instead identified a *C. parapsilosis* vacuolar-localized ZnT, CpZrc1, which is essential for zinc detoxification and also protects *C. parapsilosis* cells against killing by murine macrophages. Based on our findings, we can hypothesize that, once *C. parapsilosis* cells are phagocytosed by macrophages, zinc ions accumulate in the phagolysosome which may contribute towards fungal killing (figure 12*b*).

## Data Availability

All data are available in the manuscript or as electronic supplementary material [45].
